# 355 As it Should Be: A Tale of Antimicrobial Stewardship and Hospital Onset- C Difficile

**DOI:** 10.1017/ash.2026.10693

**Published:** 2026-06-23

**Authors:** Anika Patel, Tara Schmidt, Elizabeth Monsees, Terri Rebmann, Patricia Stone, Pamela De Cordova, Monika Pogorzelska-Maziarz

**Affiliations:** 1 Sidney Kimmel Medical College; 2 Children’s Mercy Hospital; 3 Columbia School of Nursing; 4 Rutgers, the State University of New Jersey

## Abstract

**Background:** The COVID-19 pandemic placed unprecedented strain on infection prevention and control (IPC) departments across the country. Infection Preventionists (IPs) found themselves tasked not only with sustaining routine IPC activities, but also with managing acute, crisis-driven responsibilities. The rapid increase in workload exposed long-standing gaps in staffing, communication, preparedness, and organizational support. There are limited first-hand accounts from IPC professionals and most focus on data collected during the height of the pandemic. This study examines their retrospective lessons learned to inform future preparedness. **Methods:** We analyzed responses to an open-ended item from the national 2023 AHRQ-funded Prevention of Infection Through Appropriate Staffing (PITAS) survey. Survey respondents were asked to list the three most important lessons learned from the pandemic that might help other institutions cope with future pandemics. Two study investigators performed a qualitative content analysis using an inductive-deductive approach to categorize responses into overarching themes and subthemes. Themes were then mapped to the Resilience Engineering framework (Anticipate, Monitor, Respond, Learn) and CDC Public Health Emergency Preparedness and Response (PHEPR) Capabilities to assess alignment with established public health preparedness domains. **Results:** We received 514 survey responses that mapped onto eight key themes. The most frequently cited themes emphasized the need for transparent, multi-directional communication (n = 152), especially regarding the ability to communicate frequently evolving guidance. There was emphasis on the need for adaptive, evidence-based staff education (n = 145) regarding PPE usage and pandemic preparedness. Participants also highlighted the importance of collaborative, inclusive teamwork across departments (n = 128) and the necessity of maintaining a sustainable, flexible workforce capacity (n = 123). Additional lessons focused on the value of agile policies and procedures (n = 114), comprehensive preparedness and response infrastructure (n = 113), and essential resources such as PPE and supply-chain systems (n = 99). Participants also underscored the importance of cultivating a resilient mindset and effective coping strategies (n = 100) to navigate and persevere through prolonged crisis conditions. **Conclusion:** Reflecting on the COVID-19 pandemic, IPs across the country emphasized the importance of strengthening communication systems, increasing workforce capacity, fostering cross-disciplinary collaboration, and ensuring resource reliability. Improvements in organizational infrastructure, increased leadership support, and the development of personal coping strategies will be essential for enhancing institutional resilience during future infectious disease emergencies. These lessons provide actionable guidance for supporting IPC programs and improving institutional readiness for future infectious disease emergencies.

